# A comparison between the Disposcope endoscope and fibreoptic bronchoscope for nasotracheal intubation: a randomized controlled trial

**DOI:** 10.1186/s12871-019-0834-3

**Published:** 2019-08-23

**Authors:** Junma Yu, Rui Hu, Lining Wu, Peng Sun, Zhi Zhang

**Affiliations:** 1 Hefei National Laboratory for Physical Sciences at the Microscale, Department of Biophysics and Neurobiology, University of Science and Technology of China, Hefei, 230027 People’s Republic of China; 2 Department of Anesthesiology, The First People’s Hospital of Hefei, Anhui Medical University, Hefei, Anhui 230061 People’s Republic of China

**Keywords:** Disposcope endoscope, Nasotracheal intubation, Fibreoptic, Video stylet, Endotracheal tube

## Abstract

**Background:**

Nasotracheal intubation (NTI) is frequently performed for oral and maxillofacial surgeries. This study evaluated whether NTI is easier when guided by Disposcope endoscopy or fibreoptic bronchoscopy.

**Methods:**

Sixty patients (30 per group) requiring NTI were randomly assigned to undergo fibreoptic bronchoscopy-guided (fibreoptic group) or Disposcope endoscope-guided (Disposcope group) NTI. The NTI time, which was defined as the time from when the fibreoptic bronchoscope or aseptic suction catheter was inserted into the nasal cavity to the time at which the tracheal tube was correctly inserted through the glottis, was recorded. Epistaxis was evaluated by direct laryngoscopy five minutes after completing NTI and was scored as one of four grades according to the following modified criteria: no epistaxis, mild epistaxis, moderate epistaxis, and severe epistaxis.

**Results:**

The time to complete NTI was significantly longer in the fibreoptic group than in the Disposcope group (38.4 s vs 24.1 s; mean difference, 14.2 s; 95% confidence interval (CI), 10.4 to 18.1). Mild epistaxis was observed in 8 patients in the fibreoptic group and in 7 patients in the Disposcope group (26.7% vs 23.3%, respectively; relative risk, 1.2; 95% CI, 0.4 to 3.9), though no moderate or severe epistaxis occurred in either group. Furthermore, no obvious nasal pain was reported by any of the patients at any time point after extubation (*P* = 0.74).

**Conclusion:**

NTI can be completed successfully using either fibreoptic bronchoscopy or Disposcope endoscope as a guide without any severe complications. However, compared to fibreoptic bronchoscopy, Disposcope endoscope requires less execution time (the NTI time).

**Trial registration:**

This clinical research was registered at the Chinese Clinical Trial Registry (www.chictr.org.cn, ChiCTR-IPR-17011462, date of registration, May 2017).

## Background

Nasotracheal intubation (NTI) is frequently used during oral and maxillofacial surgeries [1], and possible complications, especially epistaxis and trauma to the airway, can occur [2]. Fibreoptic bronchoscopy-guided NTI is associated with less epistaxis and better navigability and has a lower redirection rate [3]. In other studies, compared with the Macintosh laryngoscope, fibreoptic bronchoscopy-guide NTI resulted in a lower rate of sore throat and significantly shortened the total intubation time, and improved field of view during intubation and shortened intubation time were reported for the McGrath MAC laryngoscope [4, 5].

**Fig. 1:**
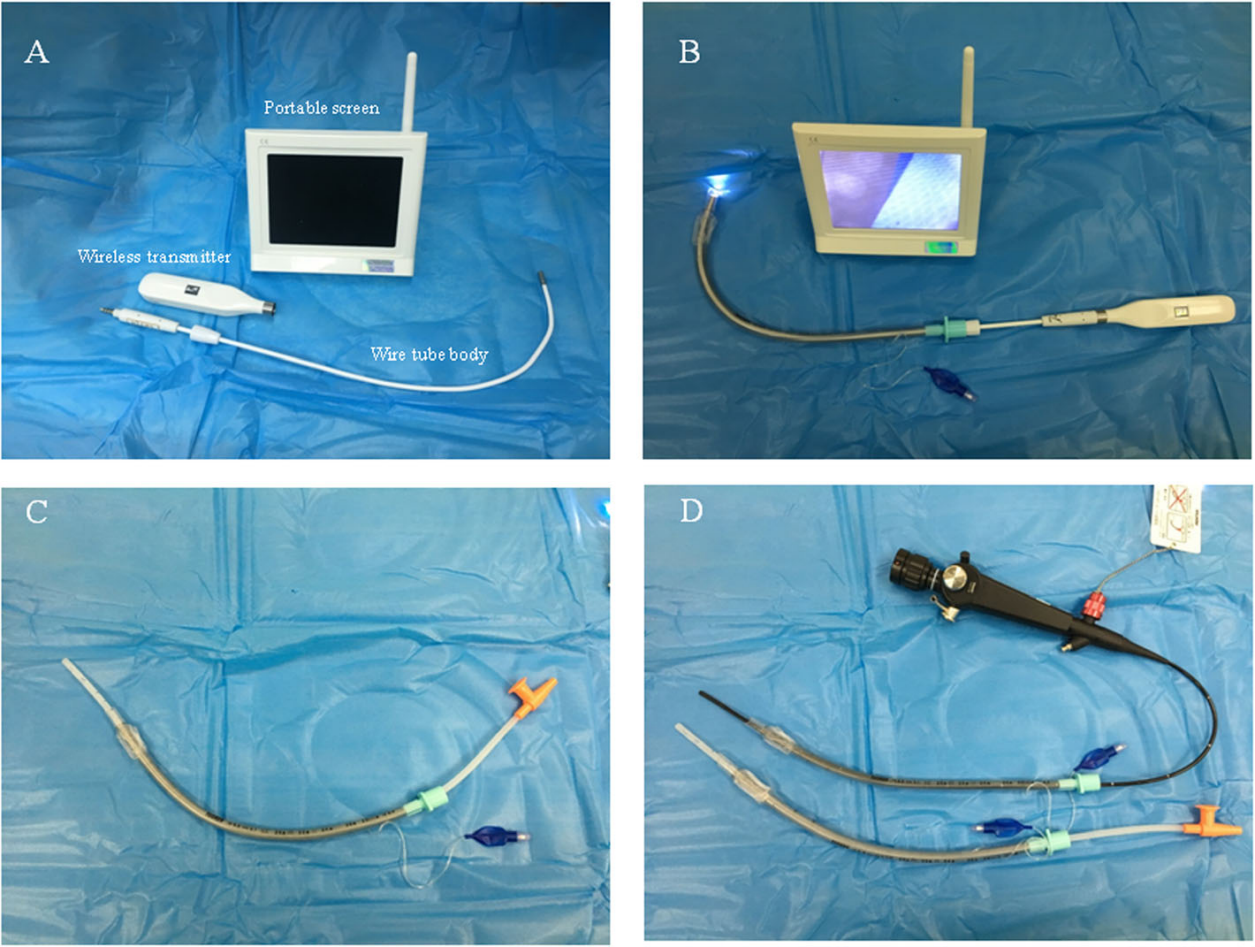
The Disposcope endoscope (Dexscope™, Yangzhou Dex Medical Device Co., Ltd., Yangzhou, China, **a**). The depth of wire transfer was pre-measured to ensure that the wire tip did not exceed the tube before NTI. (**b**) An aseptic suction catheter (OD, 5.33 mm, TUORen Medical Equipment Co., Henan, China, **c** and **d**) was inserted through the tracheal tube (TUORen Medical Equipment Co., Henan, China) and fibreoptic bronchoscope (Pentax FI-10BS, Pentax Corporation, Tokyo, Japan, **d**)

The Disposcope endoscope (Dexscope™, Yangzhou Dex Medical Device Co., Ltd., Yangzhou, China, produced in 2014) is a video stylet used for endotracheal intubation. Its wire tube body is composed of rigid metal, but it can easily be bent during surgery, enabling doctors to adjust it to the optimum angle for each patient and situation (Fig. 1, a and b). Compared to the Macintosh laryngoscope, the Disposcope endoscope yields a higher success rate for endotracheal intubation and provides a better view of the glottis; it is also associated with a shorter intubation time and causes fewer dental injuries when used to imitate intubation on a manikin wearing a semi-rigid neck collar [6]. Moreover, the Disposcope endoscope demonstrated a promising ability to guide successful endotracheal intubation in trauma patients wearing a semi-rigid neck collar [6]. Another study showed that the Disposcope endoscope can also be applied successfully in double-lumen tube placement [7].

We hypothesized that Disposcope endoscope would be as effective as fibreoptic bronchoscopy in guiding NTI.

## Methods

In a pilot study (5 patients in each group intubated by a trained anaesthesiologist who was familiar with both techniques) prior to this research, the NTI time, which was defined as the time from when the fibreoptic bronchoscope or aseptic suction catheter was inserted into the nasal cavity to the time at which the tracheal tube was correctly inserted through the glottis, was significantly longer in the fibreoptic group than in the Disposcope group (43.0 ± 13.4 s vs 24.0 ± 3.2 s). For this study, the total sample size to achieve 95% power and an α-error of 5% was 8 patients per group according to G*Power 3.1.9.4 software. Sixty adult patients rated American Society of Anaesthesiologists (ASA) I and II who were scheduled to undergo elective oral and maxillofacial surgery requiring NTI under general anaesthesia were selected. We excluded patients from our study if they fell into any of the following categories: (1) age younger than 18 years or older than 80 years; (2) a body mass index (BMI) ≥ 30 kg/m^2^; (3) a preoperative Mallampati score of III or higher; (4) a history of nasal abnormality (e.g., nasal trauma, surgery, obstruction, and polyps); (5) current anticoagulation therapy; (6) the presence of an oral malignant tumour or difficulty anticipated in airway management; (7) a mental disorder diagnosis; and (8) cervical vertebra instability, trauma or rheumatoid arthritis. None of the patients were premedicated, and standard monitoring equipment was used in the operating room. All study subjects were randomized by a researcher blinded to the study, and envelopes containing randomization numbers were used to allocate the patients to the following two groups (*n* = 30 per group) according to the airway device that would be used to guide NTI: the fibreoptic bronchoscopy-guided group (fibreoptic group) and the Disposcope endoscope-guided group (Disposcope group).

General anaesthesia was induced with 1.5–2 mg/kg intravenous propofol and 0.3 μg/kg sufentanil, and muscle relaxation was achieved by intravenous administration of 0.15 mg/kg cisatracurium. Airway size and patency were estimated by fibreoptic bronchoscopy (Pentax FI-10BS, Pentax Corporation, Tokyo, Japan) in each nostril. Before intubation, manual ventilation was performed with 100% oxygen through a facemask for 3 min. Five drops of 1% ephedrine solution were instilled into larger nasal cavities to prevent bleeding. Males and females were intubated with 6.5-mm and 6.0-mm wire-reinforced tracheal tubes, respectively (TUORen Medical Equipment Co., Henan, China) with high-volume, low-pressure cuffs. Anaesthesia was maintained with propofol and remifentanil at rates of 0.1–0.15 mg/kg/min and 0.1–0.2 μg/kg/min, respectively.

**Fig. 2:**
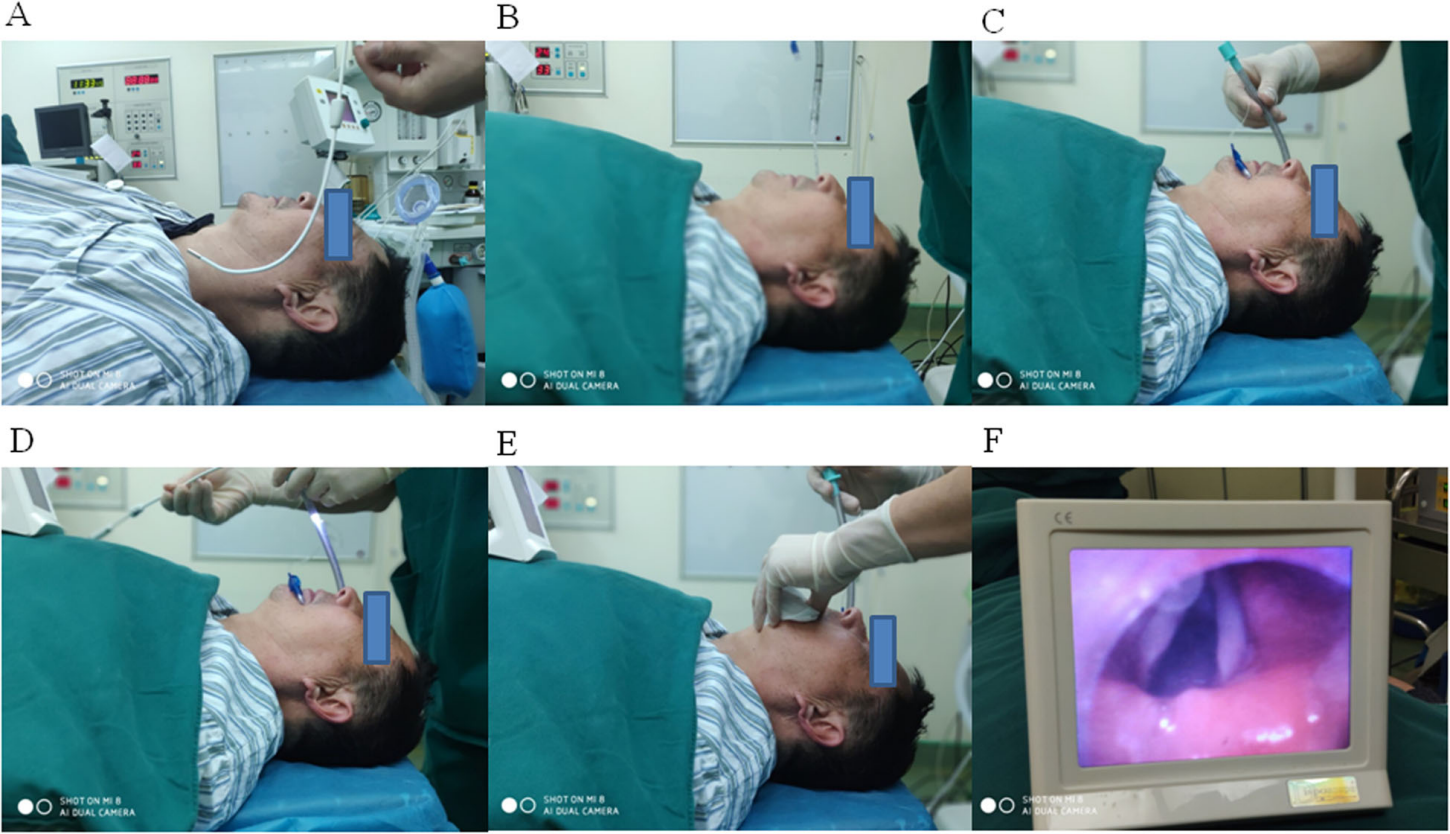
The entire process for nasotracheal intubation using the Disposcope endoscope (**a**-**f**). (**a**) The wire tube body was bent along the radian of the nasal cavity. (**b** and **c**) The tracheal tube was inserted through the nasopharynx under suction catheter guidance until the placement depth reached 15 cm. (**d**-**f**) The suction catheter was withdrawn, and NTI was then performed under the guidance of the Disposcope endoscope

In the fibreoptic group, intubation was performed with the one-hand manoeuvre by putting the little finger below the mandible angle, the ring finger below the mandible body, and the middle finger under the mental region; this gesture mimics the one-handed facemask ventilation technique. By applying this manoeuvre, the operator can simultaneously insert the fibreoptic bronchoscope and lift the chin [8]. In the Disposcope group, the depth of the wire body that was lubricated with aseptic liquid paraffin for insertion was pre-measured to ensure that the wire tip did not protrude from the tube before NTI, and the shape of the wire transfer was curved by the operator before NTI. An aseptic suction catheter (OD, 5.33 mm, TUORen Medical Equipment Co., Henan, China) lubricated with aseptic liquid paraffin was then inserted through the tracheal tube (Fig. 1, c and d). The tip of the catheter was directed ventrally with the tip of the catheter protruding from the distal end of the tube by approximately 10 cm [9], and the tracheal tube was then advanced through the nasopharynx. The suction catheter was withdrawn after the above steps were completed. The operator then used the thumb and index finger of one hand to lift the mandible during intubation [6]. The entire intubation process is shown in Fig. 2 (a-f). All intubations were performed by an anaesthesiologist who was familiar with both techniques and had 15 years of experience and a trained assistant. Minute adjustments to ventilation were performed to maintain end-tidal CO_2_ pressures at 35–45 mmHg after intubation.

The NTI time was recorded. Epistaxis was assessed by an investigator blinded to the group assignments using direct laryngoscopy at five minutes after completing NTI and was scored as one of four grades according to the following modified criteria: no epistaxis (no blood observed on either the surface of the tube or the posterior pharyngeal wall); mild epistaxis (blood apparent on the surface of the tube or posterior pharyngeal wall); moderate epistaxis (pooling of blood on the posterior pharyngeal wall); and severe epistaxis (a large amount of blood in the pharynx impeding NTI and necessitating urgent orotracheal intubation) [10].

Each patient received 0.1 μg/kg sufentanil intravenously for postoperative analgaesia upon completion of the operation. Neuromuscular blockade was reversed using neostigmine (1 mg) and atropine (0.5 mg), and the trachea was extubated when the patient was awake. At 15 min, 1 h and 24 h after extubation, the patients were asked to rate their nasal pain on a visual analogue scale (VAS) according to a 10-cm vertical score ranging from 0 = no pain to 10 = worst pain imaginable by an independent anaesthetist who was unaware of which method had been used for NTI.

The study protocol was reviewed and approved by the Institutional Research Ethics Committee of The First People’s Hospital of Hefei (No. 2016–6) on 3 March 2016. The study was also registered in the Chinese Clinical Trial Registry (www.chictr.org.cn, ChiCTR-IPR-17011462). Informed written consent was obtained from all patients in this study, and the study was conducted in accordance with the Declaration of Helsinki.

Data are expressed as the mean (SD). Parametric data were compared between the groups by analysis of variance and post hoc testing. The mean difference and the 95% confidence interval (CI) of the mean difference were calculated. Categorical data were analysed using Fisher’s exact test. The relative risks of the proportion of categorical data and 95% CIs were calculated. Statistical significance was considered at *P* values < 0.05. All statistical analyses were performed with Statistical Package for Social Sciences (SPSS) software 13.0.

## Results

**Fig. 3:**
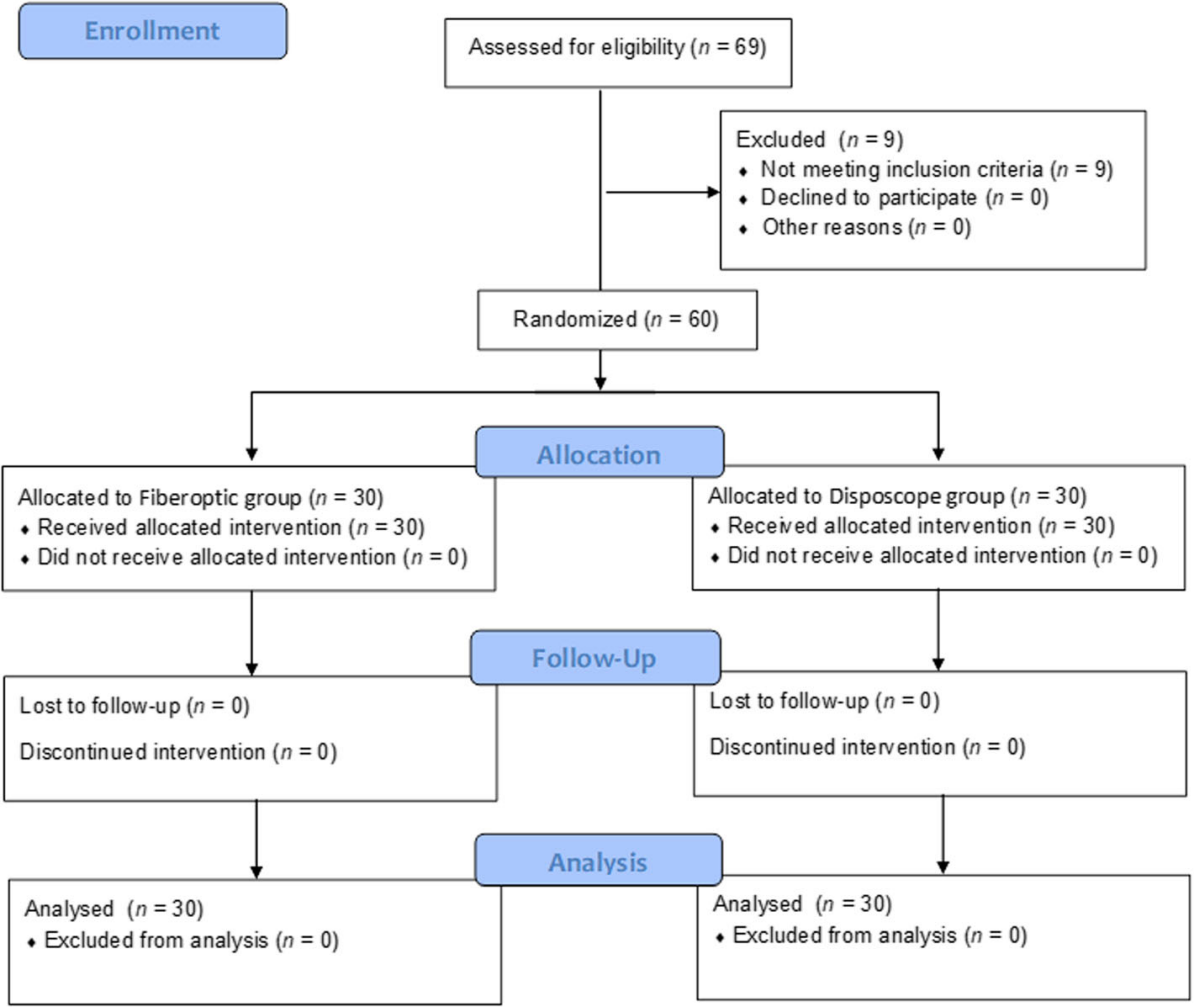
Flow chart illustrating the recruitment and loss of patients in the fibreoptic group and Disposcope group

Sixty patients consented to participate in the study. Figure 3 shows the CONSORT flow diagram for patient inclusion. No significant differences were identified between the groups with regard to patient age, height, weight, BMI, ASA score, Mallampati score, sex ratio or duration of tracheal tube indwelling time (Table 1).

**Table 1:** Patient characteristics and the duration of anaesthesia

Variable	Fibreoptic group (*n* = 30)	Disposcope group (*n* = 30)	*P* value
Age (years)	43.4 ± 15.5	47.2 ± 15.5	0.69
Height (cm)	165.6 ± 7.6	164.1 ± 7.1	0.75
Weight (kg)	63.8 ± 7.6	64.1 ± 11.0	0.21
ASA physical status (І/П)	20/10	18/12	0.59
Sex (male:female)	14/16	12/18	0.60
BMI (kg·m^−2^)	23.3 ± 2.7	23.8 ± 3.2	0.54
Mallampati score	1.5 ± 0.5	1.4 ± 0.5	0.36
Duration of anaesthesia (min)	69.1 ± 27.0	72.1 ± 29.2	0.98

Values are expressed as a number or the mean (SD)

The time to complete NTI (the NTI time) was significantly longer in the fibreoptic group than in the Disposcope group (38.4 s vs 24.1 s; mean difference, 14.2 s; 95% CI, 10.4 to 18.1) (Table 2).

**Table 2:** NTI time and epistaxis incidence in the groups

Variable	Fibreoptic group (*n* = 30)	Disposcope group (*n* = 30)	Difference in the means or relative risks (95% CI)	*P* value
NTI time (s)	38.4 ± 9.7	24.1 ± 3.9	14.2 (10.4 to 18.1)	0.01
Mild epistaxis	8/30 (26.7%)	7/30 (23.3%)	1.2 (0.4 to 3.9)	0.77
Moderate epistaxis	0	0	–	–
Severe epistaxis	0	0	–	–

Values are expressed as a number, a proportion (%) or the mean (standard deviation). Relative risks were calculated for categorical data. CI = confidence interval

Mild epistaxis (nasal bleeding) was observed in 8 patients in the fibreoptic group and in 7 patients in the Disposcope group (26.7% vs 23.3%, respectively; relative risk, 1.2; 95% CI, 0.4 to 3.9). No moderate or severe epistaxis occurred in either group (Table 2).

Furthermore, no obvious nasal pain was reported at any time point after extubation in the Disposcope group or the fibreoptic group, with no significant difference between the two groups (*P* = 0.74, data not shown).

## Discussion

Fibreoptic bronchoscopy-guided NTI is a well-established and safe technique with a high success rate and low morbidity. In this study, both fibreoptic bronchoscopy and Disposcope endoscope-guided NTI were successfully completed without any severe adverse reactions. However, less execution time was required when using the Disposcope endoscope, which is a video laryngoscope, than when using fibreoptic bronchoscopy. This is the first study in which the Disposcope endoscope was used for NTI.

Fibreoptic bronchoscopy-guided NTI is a favoured and popular procedure. For example, Shih et al. [8] reported that performing the one-hand manoeuvre not only saves time but also reduces the need for assistance in patients requiring fibreoptic bronchoscopy-guided NTI for reasons other than a difficult airway. However, these authors did suggest that the one-hand manoeuvre was not suitable for novices and that a trained assistant should always be available in cases of finger and hand fatigue or other unpredictable conditions [8]. Head tilt with chin lift techniques provides adequate airway support for patients with and without a limited mouth opening [11]. In this study, the NTI time was significantly lower in the Disposcope group than in the fibreoptic group. The main reasons were as follows. First, the wire tube body of the Disposcope endoscope is rigid but can be bent along the radian of the nasal cavity; thus, the left hand is fully available to lift the mandible to perform a chin lift. Second, in the fibreoptic group, we needed to use the left hand to facilitate insertion of the wire tube body because of its softness [8], and in most cases, we spent more time searching for the glottis.

Previous studies have discussed many strategies to reduce the risk of epistaxis during NTI in clinical practice. First, data have shown that less epistaxis occurs during NTI and that intubation is faster in the right as opposed to the left nostril, which is related to the anatomy of the structures located on the posterior nasopharyngeal wall; thus, the right nostril should be selected if patency appears to be equal on both sides of the nose [1, 12, 13]. Measurement of the nasal flow rate has also been reported to be a useful clinical strategy for selecting which nostril to use for NTI [14]. Second, using a Parker Flex-Tip tube not only helps to minimize the incidence of nasal mucosal trauma during NTI but may also increase patient safety and comfort [15]. Another study reported that using a stylet-Parker tube enhanced the ease of insertion through the nasopharynx and reduced the risk of epistaxis during NTI [16]. In contrast, Earle et al. [9] found that a Parker tube did not significantly reduce epistaxis during NTI compared to a standard tube. Therefore, stylet-Parker tubes were not used in our study. Furthermore, performing NTI under suction catheter guidance represents a simple and effective method for smoothly introducing a nasal endotracheal tube and reducing nasal bleeding during NTI [9]. In other studies, the placement of a bougie through the nasopharyngeal airway also protected the nasal mucosa, helped guide the tracheal tube and was associated with less epistaxis as well as better navigability and a lower redirection rate [3, 17]. In the present study, no moderate or severe epistaxis occurred in either group, perhaps for the following reasons: each nostril was pre-measured for size and patency by fibreoptic bronchoscopy, and five drops of 1% ephedrine solution were instilled into larger nasal cavities to prevent bleeding. NTI was also performed under the guidance of suction catheters. Overall, the use of wire-reinforced tracheal tubes and aseptic suction catheters may also decrease the incidence of epistaxis.

In the Disposcope group, a rigid wire transfer was inserted into the tube during NTI. However, in this study, no obvious nasal pain was reported at any time point after extubation in the Disposcope group, and no significant difference was found between the groups. We hypothesized that the wire-reinforced tracheal tubes may have protected the nasal mucosa and the entire nasal passage during surgery. In previous studies, a lower rate of sore throat after NTI was observed in the fibreoptic group than when using the Macintosh laryngoscope [4]. However, we did not assess the rate of sore throat in our study because some of the surgeries were on the vocal cords.

Admittedly, several limitations to our study should be considered. First, fibreoptic bronchoscopy-guided NTI is widely known for its effectiveness in patients with difficult airways. Unfortunately, patients with preoperative Mallampati scores of III or greater were excluded from our study because we aimed to evaluate two methods of guided NTI in elective oral and maxillofacial surgeries. Therefore, an additional study, which we plan to conduct, will be needed to confirm this effect. Second, only one anaesthesiologist performed all the intubations, which may reflect a limitation and possible bias in the study. Furthermore, the NTI time is probably not clinically important when the difference is only a dozen seconds, which may not indicate clear superiority but may suggest an optimal choice. Nonetheless, we consider the NTI time to be important in emergency cases, possibly shortening the first aid time.

## Conclusion

NTI can be successfully completed using fibreoptic bronchoscopy or a Disposcope endoscope as a guide without any severe complications. However, less time for NTI was required when using the Disposcope endoscope, which is a video laryngoscope, than when using fibreoptic bronchoscopy, and we consider the Disposcope necessary in emergency cases.

## Data Availability

The datasets analysed during the current study are available from the corresponding author upon reasonable request.

## Abbreviations

ASA: American Society of Anaesthesiologists
BMI: Body mass index
NTI: Nasotracheal intubation
VAS: Visual analogue scale
